# Articulated Multi-Instrument 2-D Pose Estimation Using Fully Convolutional Networks

**DOI:** 10.1109/TMI.2017.2787672

**Published:** 2018-01-15

**Authors:** Xiaofei Du, Thomas Kurmann, Ping-Lin Chang, Maximilian Allan, Sebastien Ourselin, Raphael Sznitman, John D. Kelly, Danail Stoyanov

**Affiliations:** 1 Centre for Medical Image ComputingUniversity College LondonLondonWC1E 6BTU.K.; 2 ARTORG Center for Biomedical Engineering ResearchUniversity of Bern3012BernSwitzerland; 3 Umbo Computer Vision Inc.San FranciscoCA94105USA; 4 Division of Surgery and Interventional ScienceUniversity College LondonLondonWC1E 6BTU.K.

**Keywords:** Surgical instrument detection, articulated pose estimation, fully convolutional networks, surgical vision

## Abstract

Instrument detection, pose estimation, and tracking in surgical videos are an important vision component for computer-assisted interventions. While significant advances have been made in recent years, articulation detection is still a major challenge. In this paper, we propose a deep neural network for articulated multi-instrument 2-D pose estimation, which is trained on detailed annotations of endoscopic and microscopic data sets. Our model is formed by a fully convolutional detection-regression network. Joints and associations between joint pairs in our instrument model are located by the detection subnetwork and are subsequently refined through a regression subnetwork. Based on the output from the model, the poses of the instruments are inferred using maximum bipartite graph matching. Our estimation framework is powered by deep learning techniques without any direct kinematic information from a robot. Our framework is tested on single-instrument *RMIT* data, and also on multi-instrument *EndoVis* and *in vivo* data with promising results. In addition, the data set annotations are publicly released along with our code and model.

## I. Introduction

Robotic surgery systems, such as the da Vinci^Ⓡ^ (Intuitive Surgical Inc, CA), have introduced a powerful platform for articulated instrument control in minimally invasive surgery (MIS) through tele-operation of the surgical camera and specialised dexterous instruments. The next generation of such platforms is likely to incorporate a more significant component of computer assisted intervention (CAI) system support through software, multi-modal data visualisation and analytical tools to better understand the surgical process and progress. Real-time knowledge of the instruments’ pose with respect to anatomical structures and the viewing coordinate frame is a crucial piece of information for such systems focused on providing assistive or autonomous surgical capabilities. While in principle with robotic instruments, the robot joint encoder data can be used to retrieve the pose information, in the da Vinci^Ⓡ^, the kinematic chain involves 18 joints, which is more than 2 meters long. This is challenging for accurate absolute position sensing and requires time-consuming hand-eye calibration between the camera and the robot coordinates. On cable driven systems the absolute error can be up to 1 inch, which means the positional accuracy is potentially too low for tracking applications without visual correction [1]–[3]. Recent developments in endoscopic computer vision have resulted in advanced approaches for 2D instrument detection for minimally invasive surgery. Most of these methods have focused on semantic segmentation of the image or on single landmark detection on the instrument tip, which cannot represent the full pose of an instrument or include articulation. Additional challenges to articulated tracking in surgical video are because information inferred from video directly can suffer from occlusions, noise and specularities, perspective changes and bleeding or smoke in the scene.

Image-based surgical instrument detection and tracking is attractive because it relies purely on equipment already in the operating theatre [4]. Likewise pose estimation from images has been shown to be feasible in different specialisations, such as retinal microsurgery [5]–[7], neurosurgery [8] and MIS [9]–[11]. While both detection and tracking are difficult, pose estimation presents additional challenges due to the complex articulation structure. Most image-based methods [7], [11] often extract low-level visual features from keypoints or regions to learn offline or online part appearance templates by using machine learning algorithms. Such low-level feature representations usually suffer from a lack of semantic interpretation, which means they cannot capture the high level category appearance. To improve robustness, it is possible to integrate external constraints such as surgical CAD models [10], [12] or robotic kinematics [1], [13], but the essential image-driven approach is still central to provide robust and generalisable systems.

Deep convolutional neural networks have emerged as the method of choice for various visual tasks [14]–[17]. In the past few years, it has been applied to medical image datasets and deep networks have been developed for various medical applications such as segmentation [18] or recognition tasks [19]. The methodology has been demonstrated to be effective in instrument presence detection [20] or localization [21]. Additionally, networks for semantic instrument segmentation have also been proposed and shown to be effective in real-time performance [22], [23]. In [24], the pose estimation task is reformulated as heatmap regression and is estimated concurrently with semantic instrument segmentation. However, few methods are yet able to jointly detect the instrument contour and to estimate articulation from it.

**Fig. 7.**
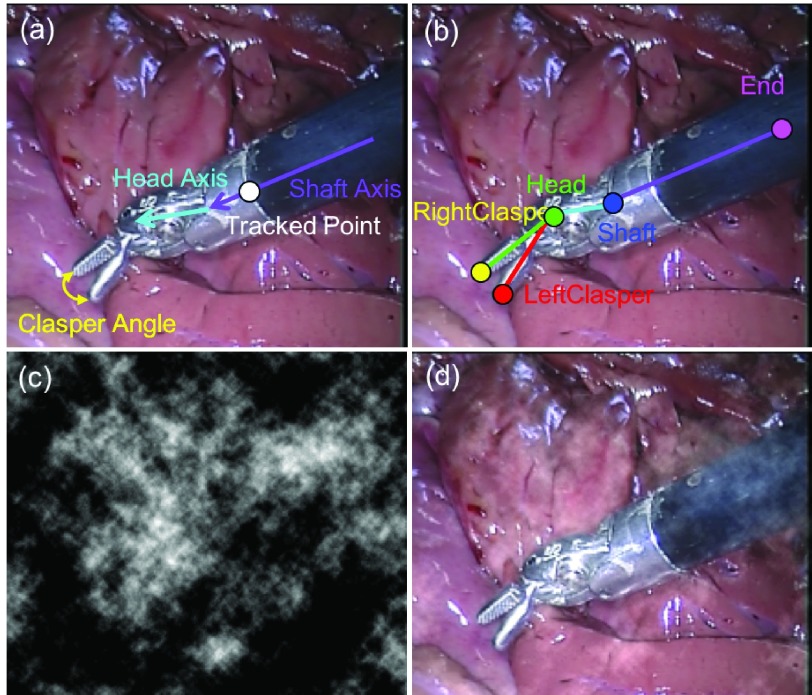
The original (a) and our proposed (b) annotation for *EndoVis* challenge dataset, smoke effect simulation (c) and simulation overlaid on the frame (d).

Following the deep learning paradigm, in this paper, we present a novel 2D pose estimation framework for articulated endoscopic surgical instruments, which involves a fully convolutional detection-regression network (FCN) and a multi-instrument parsing component. The overall scheme is able to effectively localize instrument joints and also to estimate the articulation model. To measure articulation performance, we used the single-instrument *RMIT* dataset, and we also re-annotated instrument joints of the multi-instrument dataset presented at the *EndoVis* Challenge, MICCAI’15 for training our network. Our method achieves very compelling performance and illustrates some interesting capabilities including transfer between different instrument sets within the *EndoVis* data and also between phantom settings, and *in vivo* robotic prostatectomy surgery data. The high-level of detail annotations which we have created as part of this study will naturally be made available for future research as well as our model and code (See Fig. 7).^1^^1^https://github.com/surgical-vision/EndoVisPoseAnnotation

## II. Methods

**Fig. 1.**
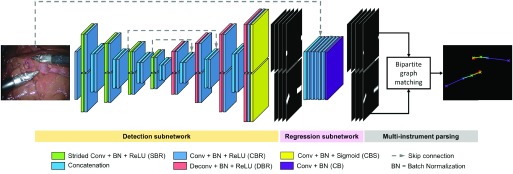
The pipeline of the proposed pose estimation framework and the detection-regression FCN architectural design. The output of the network integrates the associated joints and assembles them into the final poses for all instruments in the frame.

The overall pipeline of our deep convolutional neural network based framework is shown in Fig. 1. In this section, we first define the instrument joint structure. Then, we introduce the objective and architectural design of each module of our detection-regression FCN. In our detection-regression architecture, the detection module guides the subsequent regression module to focus on the joint parts, and the regression module helps the detection module to localize joints more precisely. Finally, we describe how the network output is integrated for inferring the poses of multiple instruments.

### A. Articulation Model Architecture

**Fig. 2.**
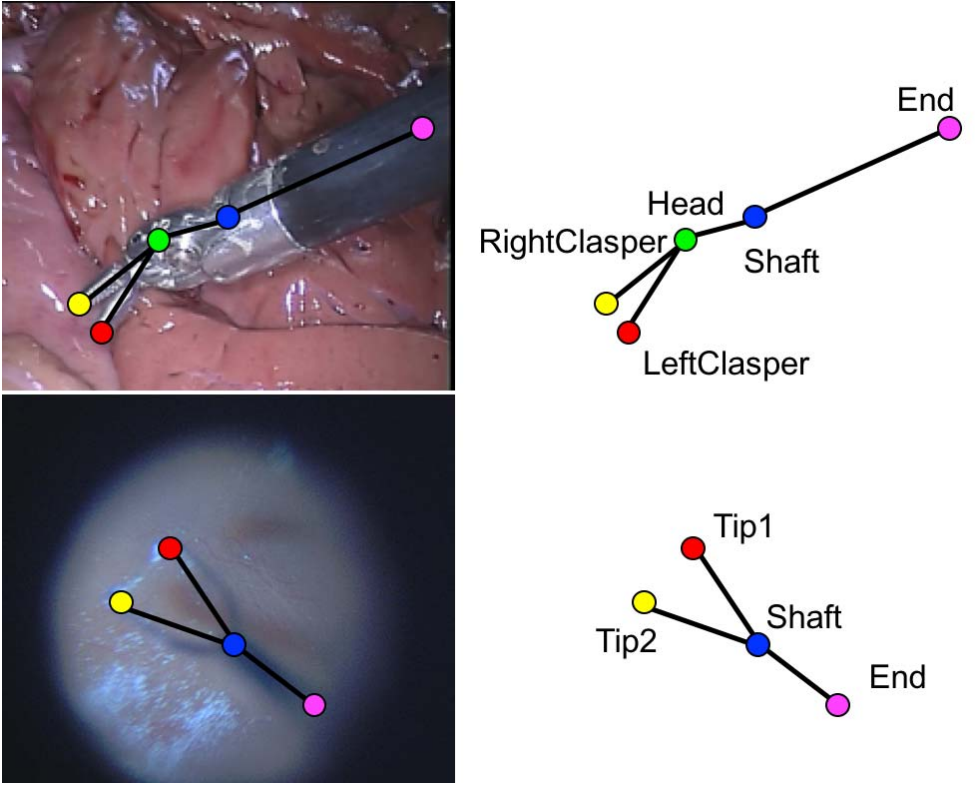
Based on the articulation, instruments in different datasets are represented with similar skeletons. They are decomposed into ${N}$ joints and ${M}$ joint pairs. Joints are represented by colour dots, and joint pairs are connected by black lines. (Top) The *EndoWrist* Needle Driver instrument is made up of 5 joints and 4 joint pairs; (Bottom) The Retinal instrument is made up of 4 joints and 3 joint pairs.

The pose of an articulated instrument can be represented in different ways. For example, it could take advantage of kinematic information by using joint relative orientation. Our work relies purely on visual cues. As shown in Fig. 2, an articulated instrument is decomposed as a skeleton of individual joint parts. We define a joint pair as two joints which are connected within the skeleton. Based on the articulation, instruments in different datasets are represented with a similar tree structure which is made up of $N$ joints and $M$ joint pairs. Therefore, the instrument pose estimation task is reduced to detecting the location of individual joint parts, and if there are multiple instruments present in the image, joints of the same instrument should be correctly associated after localization. Our bi-branch model architecture is inspired by CMUPose [15]. Joint locations and associations between joint pairs are learnt jointly via two branches of same encoder-decoder predication process. In each of the blocks, features or predictions from each branch capture different structural information about the instrument and are concatenated for the next block.

### B. Joint Detection and Association Subnetwork

We design our bi-branch joint detection and association network inspired by the recent success of FCNs [15], [18]. Since joints could overlap with each other, some pixels may belong to multiple joints. Therefore, we choose to use multiple binary cross-entropy instead of multi-class cross-entropy to train our network. By treating it as multiple binary-class problem, the ground truth we generate can reflect overlapping joint occlusion.

**Fig. 3.**
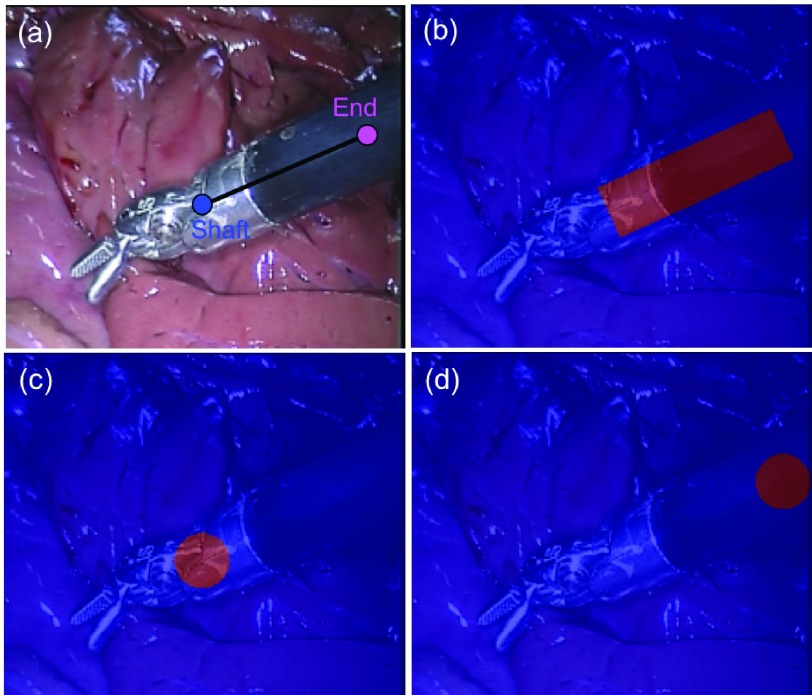
Detection subnetwork ground truth example for a Shaft-End joint pair: the binary map for Shaft-End pair association (b), the Shaft (c) and End (d) joint.

In our bi-branch network, the first branch is used to predict $N$ individual joint probability maps, one for each joint; and the second branch is used to predict the $M$ joint association probability maps, one for each joint pair. Therefore, the ground truth for the detection subnetwork is constructed as a set of $N+M$ binary maps. Similar to the original U-Net [18], we used the popular downsampling-upsampling FCN architecture. The encoder-decoder network architecture concept is widely used for semantic segmentation problems since it transfers from classification to dense pixel-wise prediction probability maps with the same size as the input image. Fully connected layers can be turned into convolution layers, which has the advantages such as reduced number of parameters, faster forward-backward pass speed or taking images of arbitrary sizes [17]. We also augmented our model with skip connections by fusing features from different layers to refine the spatial output precision. We take the Shaft-End joint pair as example, and illustrate the corresponding ground truth in Fig. 3. For joint ground truth map (Fig. 3(c–d)), the pixels located within a certain radius $r_{d}$ of the labelled location are considered as the joint, and are set to 1, and the remaining pixels are considered as background, and are set to 0. To reflect the connection relationship and to measure the association of correct joints, the association ground map is constructed as shown in Fig. 3 (b). The pixels within distance $r_{d}$ to the line connecting the joints are set to foreground, which form a rotated rectangle and are set to 1, other pixels are considered as background and are set to 0. The specifications of the network are shown in Tab. I. As shown in Fig. 1, high level encoder features are concatenated with the upsampled decoder output. Instead of pooling operations, we use strided convolution for downsampling and also eliminate fully connected layers and use all convolutional layers following the recent examples from the literature [17]. It is trained with a per-pixel binary cross-entropy loss function $L_{d}$ which is defined as:

\begin{align*}&\hspace {-2pc}L_{d}=\frac {1}{(M+N)\Omega }\sum _{k=1}^{M+N}\sum _{\mathbf {x} \in \Omega }\Big [p^{k}_{\mathbf {x}}\log \tilde {p^{k}_{\mathbf {x}}}\notag \\&\qquad \qquad \qquad \qquad \quad \qquad + \big (1-p^{k}_{\mathbf {x}}\big)\log \big (1-\tilde {p^{k}_{\mathbf {x}}}\big)\Big] \end{align*}

where $p^{k}_{\mathbf {x}}$ and $\tilde {p^{k}_{\mathbf {x}}}$ denotes the ground truth value and the corresponding sigmoid output at pixel location $\mathbf {x}$ in the frame domain $\Omega$ of the $k$th probability map.

**TABLE I:** The Network Specifications for the Detection Subnetwork: The Kernel Size and Stride, and the Output Size (Channel $\times$ Height $\times$ Width) of Each Layer. The Original Dimension of the Input Image is 3 ${\times} h \times w$, and the Network Outputs Stacked ($M+N$) Probability Maps With the Same Size as the Input Image

	Kernel (Size, Stride)	Output ($\mathrm{C}{\times} \mathrm{H}{\times} \mathrm{W}$)
Downsample		
CBR	$3\times 3,1\times 1$	$64\times h \times w$
Branch SBR1	$2\times 2,2\times 2$	$64\times {h}/{2} \times {w}/{2}$
Branch CBR1	$3\times 3,1\times 1$	$64\times {h}/{2} \times {w}/{2}$
CBR1	$1\times 1,1\times 1$	$128\times {h}/{2} \times {w}{2}$
Branch SBR2	$2\times 2,2\times 2$	$128\times {h}/{4} \times {w}/{4}$
Branch CBR2	$3\times 3,1\times 1$	$128\times {h}/{4} \times {w}/{4}$
CBR2	$1\times 1,1\times 1$	$256\times {h}/{4} \times {w}{4}$
Branch SBR3	$2\times 2,2\times 2$	$256\times {h}/{8} \times {w}/{8}$
Branch CBR3	$3\times 3,1\times 1$	$256\times {h}/{8} \times {w}/{8}$
CBR3	$1\times 1,1\times 1$	$512\times {h}/{8} \times {w}/{8}$
Branch SBR4	$2\times 2,2\times 2$	$512\times {h}/{16} \times {w}/{16}$
Branch CBR4	$3\times 3,1\times 1$	$512\times {h}/{16} \times {w}/{16}$
CBR4	$1\times 1,1\times 1$	$1024\times {h}/{16} \times {w}/{16}$
Upsample		
Branch DBR1	$2\times 2,2\times 2$	$256\times {h}/{8} \times {w}/{8}$
Branch CBR1	$3\times 3,1\times 1$	$256\times {h}/{8} \times {w}/{8}$
CBR1	$1\times 1,1\times 1$	$512\times {h}/{8} \times {w}/{8}$
Branch DBR2	$2\times 2,2\times 2$	$128\times {h}/{4} \times {w}/{4}$
Branch CBR2	$3\times 3,1\times 1$	$128\times {h}/{4} \times {w}/{4}$
CBR2	$1\times 1,1\times 1$	$256\times {h}/{4} \times {w}/{4}$
Branch DBR3	$2\times 2,2\times 2$	$64\times {h}/{2} \times {w}/{2}$
Branch CBR3	$3\times 3,1\times 1$	$64\times {h}/{2} \times {w}/{2}$
CBR3	$1\times 1,1\times 1$	$128\times {h}/{2} \times {w}/{2}$
Branch DBR4	$2\times 2,2\times 2$	$32\times h \times w$
Branch CBR4	$3\times 3,1\times 1$	$32\times h \times w$
CBR4	$1\times 1,1\times 1$	$64\times h \times w$
CBS	$1\times 1,1\times 1$	$(M+N)\times h \times w$

### C. Regression Subnetwork

From the pixel-wise prediction output of the detection network, we could obtain coarse location of each joints, but in order to obtain precise location of the joints, we add a regression network following the detection network (see Fig. 1).

The input of the network is the concatenation of the input image and the stacked $M+N$ output probability maps of the detection network, with the latter acting as a semantic guidance for the regression network to focus on the joint parts and their structural relationships. Previous work [14] showed that directly regressing single points from an input frame is highly non-linear, so instead of regressing single points, the network will produce stacked joint density maps, which have the same size as the input image. The network contains five *Conv+Batch Normalization+ReLU (CBN)* blocks, followed by a *Conv+Batch Normalization (CB)* block. The specifications of the network is shown in Tab. II.

**TABLE II:** The Network Specifications for Regression Subnetwork: The Kernel Size and Stride, and the Output Size (Channel $\times$ Height $\times$ Width) of Each Layer. The Regression Network is Fed With the Concatenation of the Input Image and the Detection Output Maps, and Outputs Stacked ($M+N$) Probability Maps With the Same Size as the Input Image

	Kernel (Size, Stride)	Output ($\mathrm{C}{\times} \mathrm{H}{\times} \mathrm{W}$)
CBR1	$3\times 3,1\times 1$	$64\times h \times w$
CBR2	$3\times 3,1\times 1$	$128\times h \times w$
CBR3	$3\times 3,1\times 1$	$256\times h \times w$
CBR4	$3\times 3,1\times 1$	$256\times h \times w$
CBR5	$1\times 1,1\times 1$	$256\times h \times w$
CB	$1\times 1,1\times 1$	$(M+N)\times h \times w$

**Fig. 4.**
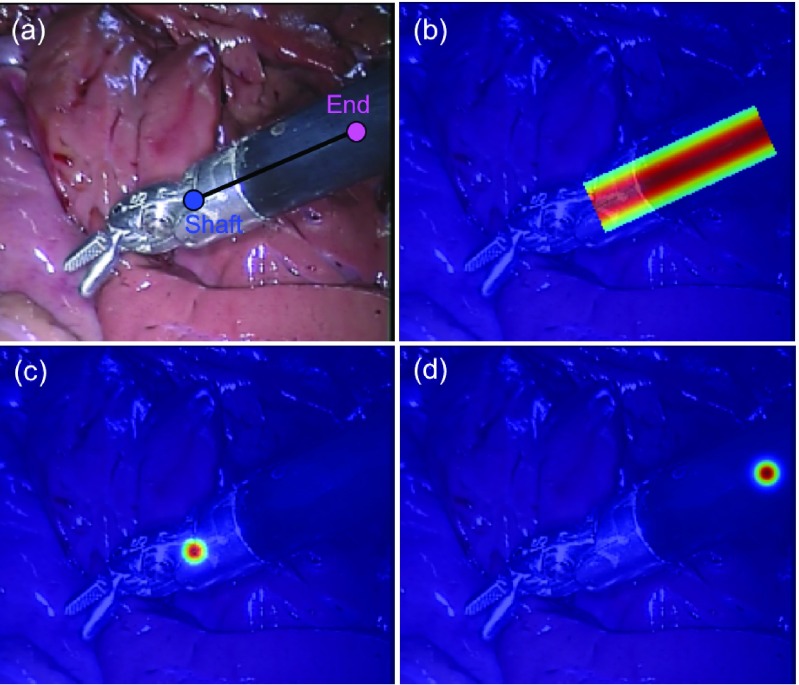
Regression subnetwork ground truth example for Shaft-End joint pair: the density map for Shaft-End pair association (b), the Shaft (c) and End (d) joint.

In Fig. 4, we illustrate the Shaft-End joint pair ground truth maps for the regression subnetwork. For joint ground truth maps (Fig. 4(c–d)), each joint annotation corresponds to an density map which is formed with a $2D$ Gaussian centred at the labelled point location. And the association ground truth density maps are represented with a Gaussian distribution along the joint pair centre line, with a standard deviation $\sigma$ shown in Fig. 4 (b). Therefore, the goal of the regression subnetwork is to regress the density maps from the input image with the guidance of the detection probability maps. It is trained with the mean squared loss $L_{r}$ which we define as:

\begin{equation*} L_{r} = \frac {1}{(M+N)\Omega }\sum _{k=1}^{M+N}\sum _{\mathbf {x}\in \Omega }\left \|{h^{k}_{\mathbf {x}}-\tilde {h^{k}_{\mathbf {x}}}}\right \|^{2} \end{equation*}

where $h^{k}_{\mathbf {x}}$ and $\tilde {h^{k}_{\mathbf {x}}}$ represent the ground truth and the predicted value at pixel location $\mathbf {x}\in \Omega$ of the $k$th density map, respectively.

### D. Multi-Instrument Parsing

After obtaining the output density maps of all the joints from the detection and regression framework, non-maximum suppression (NMS) [25] is performed on the joint density maps to obtain potential joint candidates. NMS is popularly used in deep learning and generally in computer vision to eliminate redundant candidates. It selects high-scoring candidate and skips ones that are close to an already selected candidate.

**Fig. 5.**
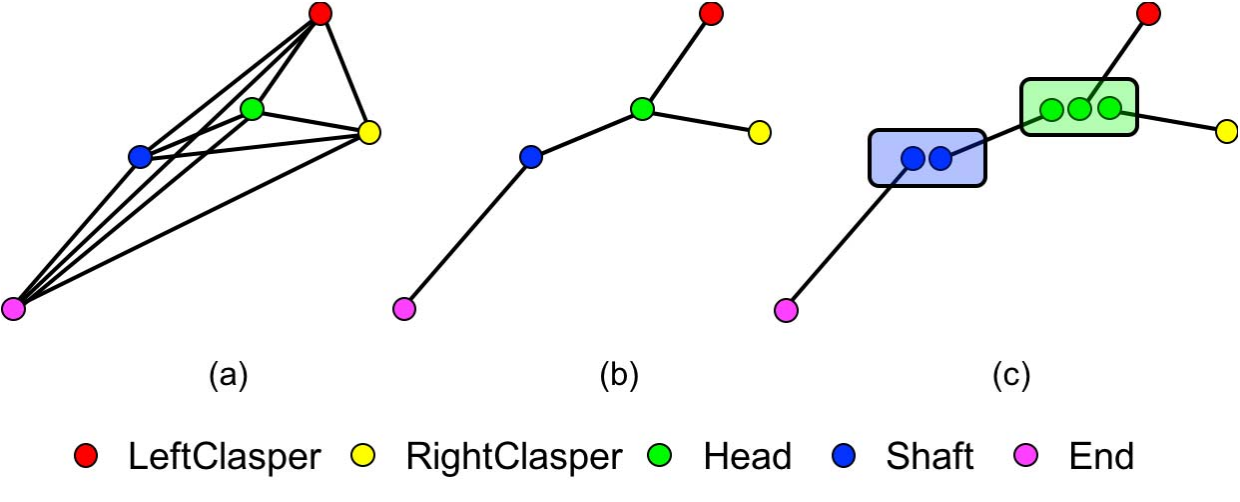
Graph relaxing for instrument structure: (a) Fully connected graph; (b) Tree structure graph; (c) A set of bipartite graphs after relaxation, the matching of joint pairs are decided independently.

As shown in Fig. 5, instead of a fully connected graph (Fig. 5(a)), where every pair is connected, the instrument structure is relaxed into a tree graph (Fig. 5(b)) with minimal number of connections. The tree graph can be further decomposed into a set of joint pairs, for which the matching is decided independently (Fig. 5(c)). The bipartite matching sub-problem then can be solved by maximum bipartite matching [26]. To eliminate outliers and connect the right joints for each instrument, the association density maps from the network output are used to measure the association of joint candidate pairs: the association score is defined as the sum of accumulated pixel values along the line connecting the joint candidates on the corresponding association density map.

The association score of any possible joint candidate pair is used to construct the weighted bipartite graphs. After finding the matching with maximum score of the chosen joint pairs, the ones which share the same joint can be assembled into full poses of multiple instruments.

## III. Experiments and Results

### A. Datasets

Our proposed pose estimation framework is evaluated on a single-instrument retinal dataset and on multi-instrument endoscopic datasets. The statistics of each dataset are summarized in Tab. III.

**TABLE III:** Label/Frame Number Summery of the RMIT and EndoVis Dataset

Seq1	Seq2	Seq3	Seq4	Seq5	Seq6	Total
$\mathit {RMIT}$ Dataset						
Train Data						
201 / 201	111 / 111	265 / 271	–	–	–	577 / 583
Test Data						
201 / 201	111 / 111	266 / 276	–	–	–	578 / 588
$\mathit {EndoVis}$ Dataset						
Train Data						
210 / 1107	240 / 1125	252 / 1124	238 / 1123	–	–	940 / 4479
Test Data						
80 / 370	76 / 375	76 / 375	76 / 375	301 / 1500	301 / 1500	910 / 4495

#### 1) Single-Instrument Retinal Microsurgery Instrument Tracking (RMIT) Dataset:

**Fig. 6.**
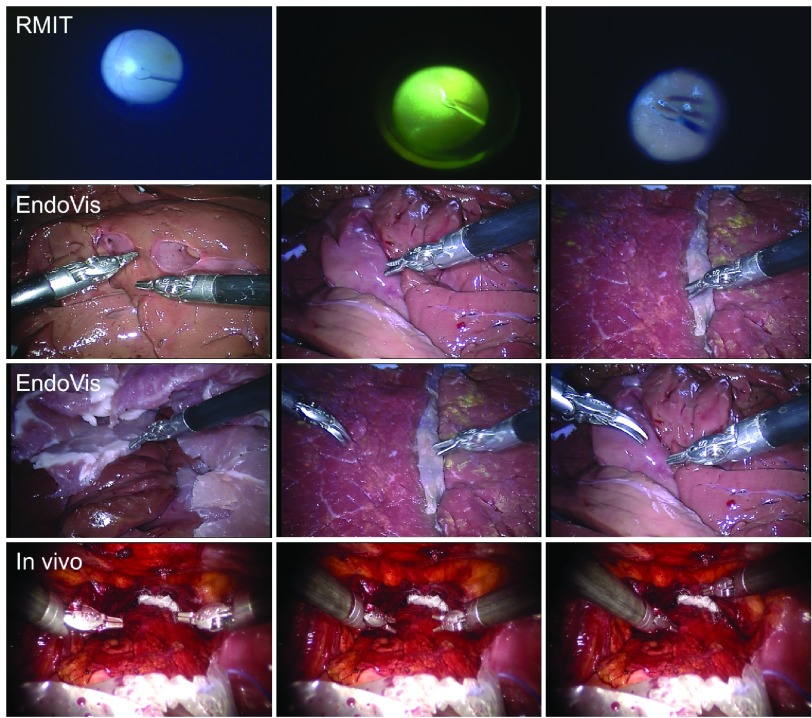
Example images from each sequence of test data in the datasets: (Top row) single-instrument Retinal Microsurgery Instrument Tracking (*RMIT*) dataset; (Middle rows) multi-instrument *EndoVis* challenge dataset; (Bottom row) multi-instrument *in vivo* dataset.

This dataset^2^ consists of three image sequences during *in vivo* retinal microsurgery, with at most a single instrument in the field of view [27] and a resolution of $640 \times 480$ pixels. The statistics of the *RMIT* dataset is summarized in the upper part of Tab. III, and frame example from each of the three sequences is shown in the top row of Fig. 6. For each sequence, four joints (Tip1, Tip2, Shaft and End Joint) of the retinal instrument are annotated for most frames. Following the same training strategy as used in previous papers [7], [27], [28], the dataset is separated into a training set including all the first halves of the sequences (577 frames), and a testing set using the second halves (578 frames).^2^https://sites.google.com/site/sznitr/code-and-datasets

#### 2) Multi-Instrument $\mathit {EndoVis}$ Challenge Dataset:

This multi-instrument dataset^3^ is separated into training and test data: the training data includes four 45 seconds *ex vivo* video sequences of interventions, the test set is composed of 15 seconds additional video sequences for each of the training sequence, and two additional 1 minute recorded interventions. The frame resolution is $720 \times 576$ pixels. Different from the original challenge guidelines, we do not enforce a leave-one-surgery-out training strategy, but use the entire training data due to our sparse annotations.^3^https://endovissub-instrument.grand-challenge.org/

The original and our proposed annotations are demonstrated in Fig. 7(a–b). The original annotation is retrieved from the robotic system, which includes the location of the intersection point between the instrument axis and the border between plastic and metal on the shaft, normalized Shaft-to-Head axis vector and the tip angle. For training and evaluating our network, we construct a high quality multi-joint annotation for this dataset. For each instrument, five joints including Left, Right Clasper, Head, Shaft and End joint are annotated. Compared to our multiple joint annotations, the original annotations only provide limited and non-intuitive pose information for training and testing purposes. We manually labelled 940 frames of the training data (4479 frames) and 910 frames for the test data (4495 frames). The label and frame number of the *EndoVis* dataset are summarized in the lower part of Tab. III, and frame examples from each sequence are shown in the middle rows of Fig. 6. It is worth mentioning that in the additional video sequences in the test set there is a *EndoWrist* Curved Scissor instrument which does not appear in the training set.

To test the performance against noise, we also add Fractional Brownian Motion noise [29] on the test data in order to simulate smoke effect during surgery (see Fig. 7(c–d)).

#### 3) Multi-Instrument ${\textit {In Vivo}}$ Dataset:

Additionally, to test the framework performance on *in vivo* data, we labelled 123 frames of video clips (1220 frames) which are obtained from robotic prostatectomy surgery conducted at University College London Hospitals NHS Foundation Trust (UCLH) with resolution of $1920 \times 1080$ pixels. Frame examples from the *in vivo* data are shown in the bottom row of Fig. 6.

### B. Training and Runtime Analysis

We implemented our framework in Lua and Torch7.^4^ The training data is augmented by horizontal and vertical flipping, and is resized to $288 \times 384$ pixels for *RMIT* data, and $256 \times 320$ pixels for *EndoVis* and *in vivo* data to fit in GPU memory. The detection radius $r_{d}$ is set to 10 pixels for *RMIT* data, and to 15 pixels for *EndoVis* and *in vivo* data. The regression standard deviation $\sigma$ is set to 20 pixels. The radius of NMS is set to equal the detection radius $r_{d}$. The network is trained on a single Nvidia GeForce GTX Titan X GPU using stochastic gradient descent (SGD) with an initial learning rate of 0.001 and momentum of 0.98. The learning rate progressively decreases every 10 epochs by 5%. The processing speed achieves 8.7 fps for videos, with the network inferencing taking 24 ms and the multi-instrument parsing step taking 89 ms.^4^http://torch.ch/

### C. Experiments

The following sections first outline the performance comparisons of our framework on the single-instrument *RMIT* dataset. Then, to understand the detection-regression architecture, we perform an ablation study and report its performance on the multi-instrument *EndoVis* dataset. Finally, we finetune the model to test the performance on the *in vivo* dataset.

#### 1) RMIT Experiments:

We trained the network with all four joints and we report performance by two different metrics: the Root-Mean-Square (RMS) distance (pixels) [27] and the strict Percentage of Correct Parts (strict PCP) [30]. The RMS distance reflects the localization accuracy of a single joint, it is evaluated as correct if the estimated joint location and the ground truth is within the threshold. Meanwhile the strict PCP estimates the localization of a joint pair and is considered correct if the distances between two connected joints are both smaller than $\alpha$ times the ground truth length of the connection pair. The evaluation results are shown in Tab. IV and Tab. V. We report the average RMS error distance, only on frames which the instruments are correctly detected (within the threshold measure). The same criteria applies for other datasets evaluated in the paper. We also compared the result against the state-of-the-art methods in Tab. VI and Tab. VII. In previous papers as listed in Tab. VI and Tab. VII, only recall score is reported. Approximate numbers are obtained through the accuracy threshold graphs from the papers, which do not provide the precise number. Analogously to previous methods, the recall score is evaluated by means of threshold measure (15 pixels) for the separate joint of the pose predictions and $\alpha$ for strict PCP is set to 0.5.

**TABLE IV:** Quantitative Results of the RMIT Dataset: Precision and the Distance Error Between Ground Truth and the Estimate of Each Joint. The Threshold is Set to 15 Pixels for the Original Resolution of ${640} \times {480}$ Pixels

Recall (%) / Precision (%) / Distance (px) of the $\mathit {RMIT}$ Dataset ($Thres=15$ px)				
Tip1	Tip2	Shaft	End	Total
Train set				
100.0 / 100.0 / 2.14	100.0 / 100.0 / 2.28	100.0 / 100.0 / 1.72	100.0 / 100.0 / 2.38	100.0 / 100.0 / 2.13
Test set				
99.13 / 99.13 / 5.26	97.58 / 97.58 / 4.61	94.12 / 94.12 / 4.93	86.51 / 86.51 / 4.68	94.33 / 94.33 / 4.87

**TABLE V:** Quantitative Results of the RMIT Dataset: the Strict PCP Score of the Estimate of Each Joint Pair

Recall (%) / Precision (%) for Strict PCP of the $\mathit {RMIT}$ Dataset ($\alpha = 0.5$)			
Tip1-Shaft	Tip2-Shaft	Shaft-End	Total
Train set			
100.0 / 100.0	100.0 / 100.0	100.0 / 100.0	100.0 / 100.0
Test set			
99.13 / 99.13	97.58 / 97.58	94.12 / 94.12	96.94 / 96.94

**TABLE VI:** Quantitative Recall Performance Comparison With the State-of-the-Art Methods on the RMIT Test Set^5^

The Recall Score of the $\mathit {RMIT}$ Test Set ($Thres=15$ px)					
	Tip1	Tip2	Shaft	End	Total
DDVT [27]	–	–	< 85.0	–	–
POSE [28]	–	–	88.9	–	–
RTOA [7]	–	–	94.3	–	–
SRNet [31]	98.6	94.1	96.2	91.2	95.0
Proposed	99.1	97.6	94.1	86.5	94.3

^5^ To maintain notation consistency, the Shaft and End joint in our paper correspond respectively to End Shaft and Start Shaft joint in previous papers.

**TABLE VII:** Quantitative Strict PCP Score Comparison With the State-of-the-Art Methods on the RMIT Test Set

The Strict PCP Score of the $\mathit {RMIT}$ Test Set ($\alpha =0.5$)				
	Tip1-Shaft	Tip2-Shaft	Shaft-End	Total
POSE [28]	$\approx 95.0$	$\approx 90.0$	–	–
Proposed	99.13	97.58	94.12	96.94

**Fig. 8.**
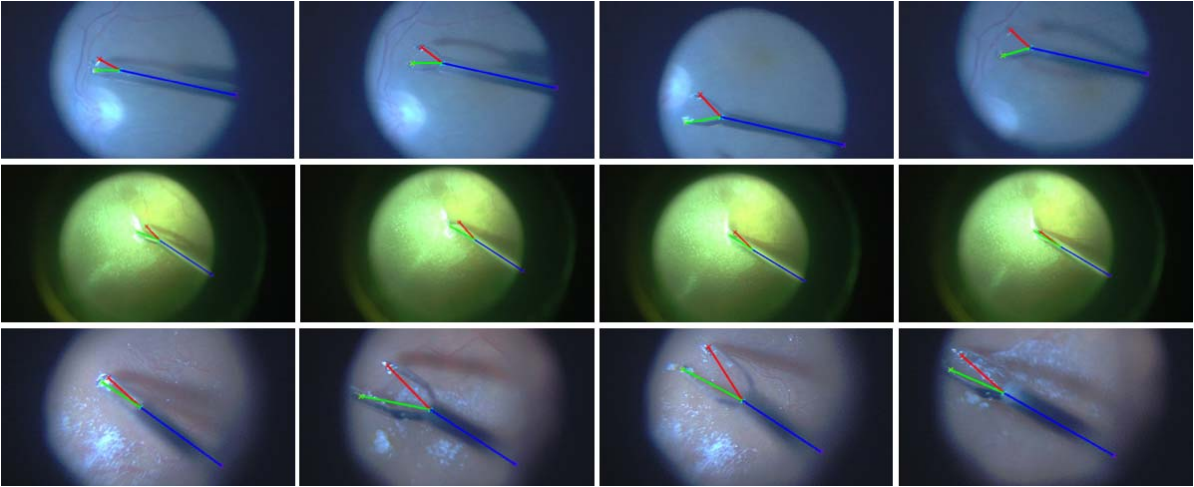
Result examples of *RMIT* test set with our model. The frame is trimmed around the instrument for better demonstration. It is difficult to localize some joints due to its ambiguous annotation, image blur or specularities.

For the proposed methods, the average joint distance error for the test set is 4.87 pixels with the same recall and precision score of 94.33%, and the average strict PCP recall score is 96.94%. Some of the test set results are shown in Fig. 8. Even under different lighting conditions, the model can predict the pose of the instrument correctly. It is interesting to point out that even though the association map used is constructed using a straight line, it still works on titled instruments (see the bottom line of Fig. 8 for example). This implies that the rectangle association maps are learnt to indicate the connection relationships between joint pairs. The trained network predict joint pair connections by not only relying on the instrument pixels, but also on the learnt joint relations and spatial contextual information. As we listed in Tab. VI, previous methods mainly focus on the evaluation of Shaft joint, except for SRNet [31], where our performance is on par with SRNet. The recall score of the End joint is the lowest (86.51%) among the four joints, due to its ambiguous annotation and image blur. SRNet uses a different strategy by explicitly modelling the instrument joints and their presence, which simultaneously predicts the instrument number and their pose. By assuming a known maximum number of instrument in the field of view, it bypasses the joint detection and association two-stage process, so can be trained in an end-to-end fashion. Adding prior could help constrain the problem, compared to SRNet, we want to treat the task as general as possible, so our model does not rely on any prior knowledge of the number of instrument, theoretically it can predict pose of arbitrary number of instrument, which one of the potential strengths of our framework.

#### 2) ${\textit {In Vivo}}$ Experiments:

Since our annotation is limited, we used our network with five joints using all the training data generated from high quality our annotation. First, we perform an ablation study^6^ to understand the detection-regression architecture. In Tab. VIII the average precision, recall score and RMS distance (pixels) of each joint for all the test data are reported. With a threshold of 20 pixels for the original resolution of $720 \times 576$ pixels, the average joint distance error for the test data set is 6.96 pixels with a recall score of 82.99% and a precision score of 83.70%.^6^An ablation study refers to evaluating how the performance is affected by removing some part of the model.

**TABLE VIII:** Quantitative Results of the EndoVis Dataset: Precision and the Distance Error Between Ground Truth and the Estimate of Each Joint. For the $\mathit {EndoVis}$ Dataset, the Thresholds are Set to 20 and 30 Pixels for the Original and Smoke-Simulated Test Data With the Resolution of 720 $\times$ 576 Pixels

Recall (%) / Precision (%) / Distance (px) of the $\mathit {EndoVis}$ Dataset					
LeftClasper	RightClasper	Head	Shaft	End	Total
Train set ($Thres=20$ px)					
100.0 / 99.95 / 2.43	100.0 / 99.95 / 2.53	99.57 / 99.68 / 2.34	100.0 / 99.95 / 2.74	99.89 / 99.84 / 6.73	99.89 / 99.87 / 3.36
Test set ($Thres=20$ px)					
86.28 / 86.65 / 5.03	85.49 / 85.82 / 5.40	75.82 / 76.81 / 6.55	90.55 / 91.50 / 8.63	76.81 / 77.71 / 9.17	82.99 / 83.70 / 6.96
Test set ($Thres=30$ px)					
89.29 / 89.67 / 5.57	87.86 / 88.19 / 5.99	80.05 / 80.99 / 7.37	94.51 / 95.42 / 9.38	89.78 / 90.68 / 11.58	88.30 / 88.99 / 7.98
Smoke Test set ($Thres=20$ px)					
83.85 / 83.48 / 5.25	82.69 / 82.27 / 5.72	74.89 / 75.07 / 6.50	89.73 / 89.71 / 8.62	82.25 / 82.55 / 8.86	82.68 / 82.62 / 6.99
Smoke Test set ($Thres=30$ px)					
88.30 / 88.02 / 6.13	86.81 / 86.41 / 6.68	78.02 / 78.30 / 7.19	95.66 / 95.66 / 9.76	91.32 / 91.48 / 10.60	88.02 / 87.97 / 8.07

In the ablation experiment, we compared the performance of five different models, including detection-only, shallow regression-only, deep regression-only, single-branch detection-regression and our proposed bi-branch detection-regression model. For the detection-only model, we use the output probability maps from the detection subnetwork for direct pose estimation. We also trained two regression-only models, a shallow one with the same architecture as the regression submodule in our detection-regression model and the input is the RGB frame without the detection probability maps, the deep one whose architecture is the same as the detection-only model and with Gaussian regression ground truth. For the single-branch model, we fuse two branches of the detection submodule into only one branch with double size of the feature maps of our model. The performance comparison of different models is summarized in Tab. IX. The bad performance of the detection-only model (32.19% / 14.41% for recall and precision score) is expected. As seen from the ground truth binary map in Fig. 3, the pixels belonging to the joint have the same weight, which lead to bad localization of joints. We also observe that both regression-only models have better performance. It is interesting that the precision score for deep model (97.67%) is higher than that for the shallow model (71.53%), while either shallow or deep regression-only models achieve similar recall performance (66.46% for shallow model and 65.06% for the deeper model). Deeper architecture does not help to achieve better recall performance in the experiment. We infer that one of the reasons is that the size of the training data is relatively small, which affects model generalization. The regression-only models are capable of predicting the location of joints without any guidance. However, regression is empirically too localized, which supports small spatial context [14], the process of regressing from original input image to joint location directly can be difficult. By combining detection and regression, the detection module guides where to focus and provides spatial contextual information between joints for the regression module, by using the probability output from the detection module as structural guidance, the regression module facilitates the detection module to localize the joints more precisely. The performance of both detection-regression models show the improvement, and furthermore, our network takes less time to train compared to regression-only model. The single-branch model achieves the performance of 76.77% / 86.16% for recall and precision, which is nearly as good as the bi-branch model. We would like to point out that single-branch and bi-branch models are essentially similar. We choose bi-branch architecture here to conceptually separate the training of joint and joint association into two branches.

**TABLE IX:** Ablation Study of the Detection-Regression Model Architecture on EndoVis Test Set

Recall (%) / Precision (%) / Distance (px) of the $\mathit {EndoVis}$ Test Set ($Thres=20$ px)					
LeftClapser	RightClasper	Head	Shaft	End	Total
Detection-only Network					
32.58 / 14.28 / 7.94	24.51 / 11.05 / 6.22	29.40 / 13.19 / 6.75	40.27 / 18.03 / 8.87	34.18 / 15.49 / 7.57	32.19 / 14.41 / 7.47
Shallow Regression-only Network					
67.73 / 72.94 / 4.86	81.26 / 84.49 / 4.34	66.48 / 73.35 / 6.18	75.16 / 80.65 / 7.58	41.65 / 46.23 / 9.06	66.46 / 71.53 / 6.41
Deep Regression-only Network					
65.75 / 98.79 / 3.63	61.81 / 93.35 / 3.80	66.48 / 99.34 / 5.12	66.65 / 99.40 / 6.84	64.62 / 97.47 / 7.15	65.06 / 97.67 / 5.31
Single-branch Detection-Regression Network					
78.90 / 88.13 / 4.70	81.04 / 90.27 / 5.44	74.07 / 83.74 / 7.24	79.56 / 88.94 / 7.72	70.27 / 79.71 / 9.22	76.77 / 86.16 / 6.87
Proposed Bi-branch Detection-Regression Network					
86.28 / 86.65 / 5.03	85.49 / 85.82 / 5.40	75.82 / 76.81 / 6.55	90.55 / 91.55 / 8.63	76.81 / 77.71 / 9.17	82.99 / 83.70 / 6.96

**Fig. 9.**
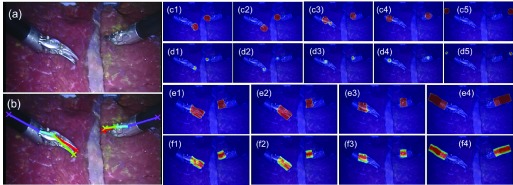
Result examples with an unseen *EndoWrist* Curved Scissor instrument in the *EndoVis* test set with our model. (a) The original frame; (b) the estimated pose; joint (c1-5) and association (e1-4) probability output from detection subnetwork; joint (d1-5) and association (f1-4) density output from regression subnetwork.

**Fig. 10.**
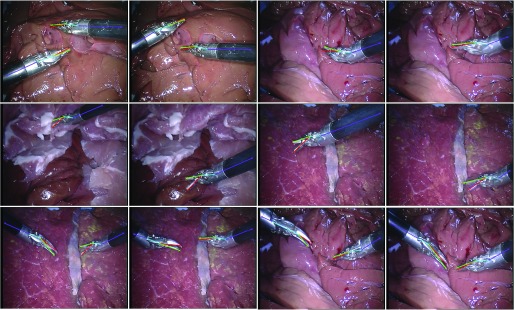
Examples of original *EndoVis* test set. Our network is able to detect a new instrument that is not present in the training data.

Similar to the *RMIT* dataset result, the lower score for the End joint (76.81% / 77.71%) is reasonable since it does not have distinct features and even the manual annotation has high variance. If the threshold is relaxed to 30 pixels, the recall and precision score of the End joint increase to 89.78% and 90.68% respectively. For the Head joint with the lowest recall and precision (75.82% / 76.81%), as we have mentioned before, the two additional sequences of the test dataset exhibit a Curved Scissor instrument which is not seen in the training set. In Figs. 9 and 10, we show some pose estimation examples from the test set. We observe that our model works well on self occlusion, as shown in the first row of Fig. 10. This is credited to: 1) the model learns the spatial relationship between joints, even if a joint is occluded, it can be inferred from other joints; 2) the training data contains self occlusion examples that can be used by the model for handling self occlusion. As we can see, the left *EndoWrist* Curved Scissor instrument has a different shape compared to the right *EndoWrist* Needle Driver instrument, which explains the relatively low score for the Head joint. But our model is general enough to detect individual parts of this new instrument. Clearly, the generalisation to an unseen new instrument is limited to certain degree. Although the left Curved Scissor instrument has different appearance, it shares the same joint configuration with the Needle Driver instrument. The results we display show that with limited training data, our model is still capable of generalising to some degree.

**Fig. 11.**
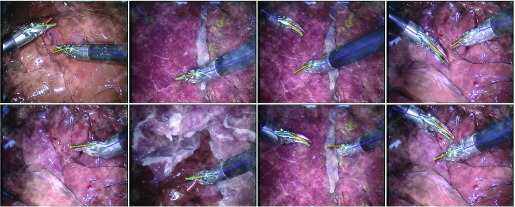
Examples of smoke-simulated *EndoVis* test set. Our network is able to detect instruments which are not seen in the training data even under smoke simulation.

From Fig. 11 and Tab. VIII we can also see that under smoke simulations the performance on test data only decrease slightly to 82.68% for recall and 82.62% for precision, with distance errors of 6.99 pixels. Please see the supplementary video for more qualitative results.

**Fig. 12.**
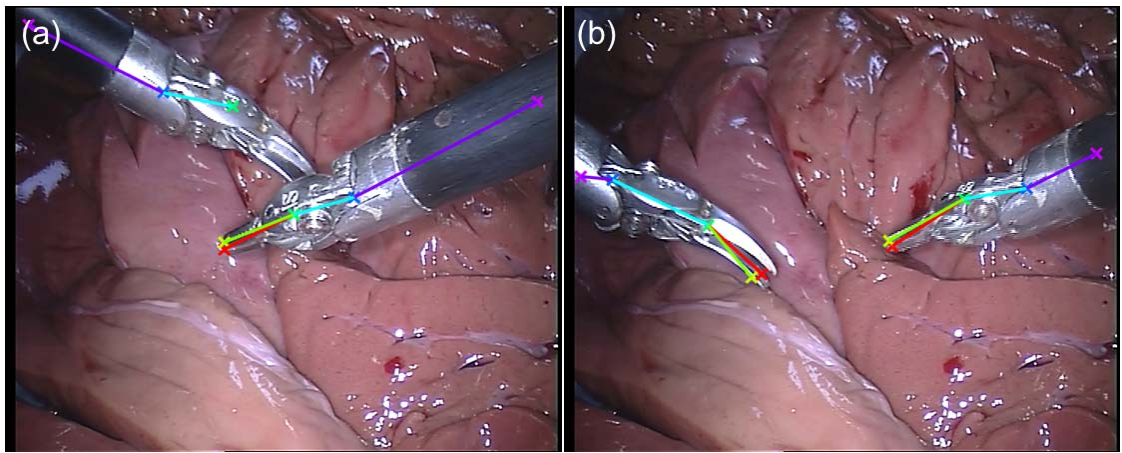
Examples of failure cases of *EndoVis* test set. (a) Occluded joints are miss detected; (b) The head joint of the new Curved Scissor instrument on the left is not well localized.

In Fig. 12, we have presented two failure cases on the test set. When one instrument is occluded by another one (Fig. 12 (a)), the model can not infer the occluded joints, we think it is due to the lack of training data on instrument overlap, which causes the model fail to learn or handle the complex situation. We can compare this to the self-occlusion (first row of Fig. 10). Since the training data covers self-occlusion, the model can well detect the self-occluded joints. We also show in Fig. 12 (b) that some joints of the new Curved Scissor instrument are not well localized, e.g. the Head joint. Our model has extended certain generalizability to unseen instrument, but obviously compared to the Needle Driver instrument in the training data, the performance is less robust.

#### 3) ${\textit {In Vivo}}$ Experiments:

**Fig. 13.**
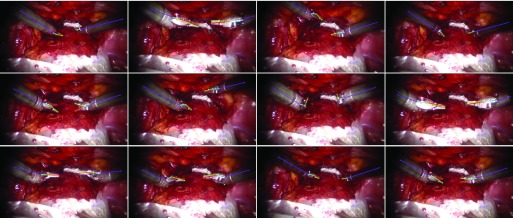
Examples of *in vivo* data with our fine-tuned model. The results demonstrate the capacity of our framework to be applied to real surgical scenes.

We fine-tuned the *EndoVis* trained model on 80% of the labelled data (97 frames) with a fixed learning rate 0.0001 for 10 epochs, and tested on the whole sequence. The *in vivo* video sequence we use is with high resolution $1920\times 1080$ pixels, so we set the threshold as 50 pixels for evaluation. In Tab. X, it is shown that the average distance errors are reduced to 9.81 and 13.42 pixels for the train and validation set respectively, with the threshold of 50 pixels for the original resolution. Examples of the *in vivo* data are shown in Fig. 13 and the pose estimation of the whole video is also included in our supplementary material. Note that we did not perform any temporal processing in any of our results.

**TABLE X:** Quantitative Results of the In vivo Dataset: Precision and the Distance Error Between Ground Truth and the Estimate of Each Joint. For the In vivo Data, the Threshold is Set to 50 Pixels for the Original Resolution of ${1920} \times {1080}$ Pixels

Recall (%) / Precision (%) / Distance (px) of the $\textit {In vivo}$ Dataset ($Thres=50$ px)					
LeftClasper	RightClasper	Head	Shaft	End	Total
Train set					
97.94 / 96.39 / 7.84	97.94 / 96.39 / 8.40	100.0 / 98.97 / 9.61	100.0 / 100.0 / 10.39	98.97 / 98.97 / 12.81	98.97 / 98.14 / 9.81
Validation set					
98.08 / 96.15 / 13.91	94.23 / 92.31 / 12.54	96.15 / 94.23 / 12.01	100.0 / 100.0 / 13.86	92.31 / 92.31 / 14.77	96.15 / 95.00 / 13.42

## IV. Conclusion

In this paper, we have proposed a deep neural network based framework for 2D pose estimation of multiple articulated instruments in surgical images and video. The methodology performs detection of the instruments and their degrees of freedom without using kinematic information from robotic encoders or external tracking sensors. The work, to the best of our knowledge, represents a novel attempt to perform image-based articulated pose estimation at this level of detail and can potentially be extended to handle even more complicated flexible articulation by incorporating additional joint nodes.

In our approach, joints and the associations between joint pairs are first detected and then refined in a detection-regression FCN. To obtain the final pose of all the instruments in an image, association probabilities are used as a measurement to connect joint pairs for each instrument by maximum bipartite matching. The framework has been trained and evaluated on *RMIT*, *EndoVis* and *in vivo* datasets with detailed annotations adding to existing challenge data labels. Interestingly, our experiments show that our model exhibits some generalizability to new unseen instrument, and has good robustness under smoke simulation. The performance on the *in vivo* datasets demonstrates the capacity of our framework to handle real surgical scenes. Our model will be publicly released to support research in the field.

A current limitation of our method is that it is limited to 2D inference and a natural extension would be to explore the estimation of 3D articulation. This seems plausible when using stereo configurations which are available within the *EndoVis* data for example and can potentially be used to formulate both the detection and the pose estimation in a joint space of both views. Additionally, it will be interesting to explore the sequential tracking of articulated instruments. This could potentially be achieved by probing the motion information that can be learnt through recurrent neural networks.
